# The Validity of Steady-State Visual Evoked Potentials as Attention Tags and Input Signals: A Critical Perspective of Frequency Allocation and Number of Stimuli

**DOI:** 10.3390/brainsci10090616

**Published:** 2020-09-07

**Authors:** Lu Wang, Dan Han, Binbin Qian, Zhenhao Zhang, Zhijun Zhang, Zhifang Liu

**Affiliations:** 1 Department of Psychology and Behavioral Sciences, Zhejiang University, Hangzhou 310028, China; 11739012@zju.edu.cn (L.W.); handan931124@zju.edu.cn (D.H.); 21939005@zju.edu.cn (B.Q.); zngzen28@gmail.com (Z.Z.); 2 Department of Psychology and Special Education, Hangzhou Normal University, Hangzhou 311121, China; liuzhifang@hznu.edu.cn

**Keywords:** steady-state visual evoked potential, attention tag, brain–computer interface, stimulus frequency, the number of stimuli

## Abstract

Steady-state visual evoked potential (SSVEP) is a periodic response to a repetitive visual stimulus at a specific frequency. Currently, SSVEP is widely treated as an attention tag in cognitive activities and is used as an input signal for brain–computer interfaces (BCIs). However, whether SSVEP can be used as a reliable indicator has been a controversial issue. We focused on the independence of SSVEP from frequency allocation and number of stimuli. First, a cue–target paradigm was adopted to examine the interaction between SSVEPs evoked by two stimuli with different frequency allocations under different attention conditions. Second, we explored whether signal strength and the performance of SSVEP-based BCIs were affected by the number of stimuli. The results revealed that no significant interaction of SSVEP responses appeared between attended and unattended stimuli under various frequency allocations, regardless of their appearance in the fundamental or second-order harmonic. The amplitude of SSVEP suffered no significant gain or loss under different numbers of stimuli, but the performance of SSVEP-based BCIs varied along with duration of stimuli; that is, the recognition rate was not affected by the number of stimuli when the duration of stimuli was long enough, while the information transfer rate (ITR) presented the opposite trend. It can be concluded that SSVEP is a reliable tool for marking and monitoring multiple stimuli simultaneously in cognitive studies, but much caution should be taken when choosing a suitable duration and the number of stimuli, in order to achieve optimal utility of BCIs in the future.

## 1. Introduction

Steady-state visual evoked potential (SSVEP) is an elapsed period of electroencephalography (EEG) activity produced by constant-frequency visual stimulation [1,2]. For example, when a 5 Hz (f refers to frequency) flicker stimulus is presented, through the Fourier transform (FFT), some specific peaks at fundamentals (f = 5 Hz) and harmonics (2f = 10 Hz, 3f = 15 Hz, and so on) can be observed in the EEG frequency domain plot. The study of SSVEP was first conducted for the information processing of luminance, largely for the measurement of spatial acuity and contrast sensitivity, where the paradigm was referred to as sweep visual evoked potential [3]. In previous studies, SSVEP has mainly been used in two fields: one is cognitive study, generally as an attention tag; while the other is for brain–computer interfaces (BCIs), chiefly as a signal source of its inputs.

Attention is an essential and enormous field in cognitive psychology, which is closely related to a great deal of mental processes such as perception, memory, thinking, and so on. Effective attention measurement can help to understand the mechanisms of attention. As an attention tag, SSVEP has been widely adopted in feature attention [4,5], working memory [6,7], binocular competition [8,9], facial processing [10,11], biological motion [12,13], and so on. Usually, SSVEP includes fundamental frequency components to examine low-level visual processing, harmonic frequency components to characterize high-level cognitive processing, and intermodulation frequency components to measure the interaction of the nervous system [14]. Compared to traditional behavioral and neuroimaging indicators, SSVEP can measure attention directly and continuously [15]. Critically, SSVEP can not only mark multiple objects with different frequencies at the same time, in order to detect dynamic attention mechanisms [16,17], but can also separate the parts from the whole, in order to independently explore the neural mechanisms of different processes [12,18]. The cognitive activities characterized by SSVEP are implicit and without explicit behavior. Therefore, SSVEP can also be applied to investigate the cognitive processing mechanisms of infants or other groups with some incapacities [19,20]. 

Although SSVEP has been widely used in cognitive studies, whether it is a reliable indicator has been a controversial issue. Similar to reaction time and other event-related potentials (ERPs), most applications of SSVEP related to cognition are based on some hypotheses. As mentioned before, simultaneously marking and monitoring multiple stimuli are salient advantages of SSVEP over other indicators. However, these advantages depend on the untested hypothesis that SSVEPs at different frequencies can be independent when multiple stimuli are simultaneously tagged; that is, there may be no mutual influence or coupling among SSVEPs at different frequencies. 

Wu and Yao (2007) compared SSVEPs under two conditions, where two stimuli were presented separately or simultaneously at flickering frequencies of 8.3 and 20 Hz [21]. Their results showed that the SSVEP responses to different frequencies were independent when the two stimuli were presented simultaneously. Nevertheless, SSVEP has a complex non-linear relationship with stimulus frequency [22], and the independence of these two specific frequencies cannot be directly extended to other frequencies. Collectively, to address this question, we first aimed to examine the hypothesis of the independence of SSVEP systematically, based on various frequency allocations. 

A BCI was designed for disabled people with severe central nervous system diseases, in order to facilitate them being able to communicate with the outside world [23]. Meanwhile, for healthy people, games controlled by a BCI system has been an attractive pattern to control a computer without movement [24,25]. At present, SSVEP is commonly adopted as an input signal for BCI, due to its higher signal-to-noise ratio (SNR), higher information transfer rate (ITR), and less user training demand [26,27]. In fact, the modulation of attention on SSVEP was a critical basis for SSVEP-based BCIs. When a participant paid attention to a stimulus flickering at a specific frequency, the corresponding SSVEP amplitude would be increased and, then, the attended stimulus could be identified through a certain algorithm. SSVEP-based BCIs, thereby, are helpful in realizing the intention of motion control or text input for users [28].

In a typical SSVEP-based BCI, a series of flicker inputs at different frequencies are always presented simultaneously to users, such that the number of stimuli may exert some additional influences. First, the number of stimuli affects their frequency intervals. It has been widely accepted that the sensitivity of SSVEP differs in different frequency bands [29]. In consideration of refresh rate of a typical LCD screen, there are limited options, in terms of the frequencies available to researchers to avoid the overlap between fundamental and harmonic frequencies [30]. To implement multiple inputs in a limited frequency range, the frequency interval must be shortened or given more concern. Hwang et al. (2012) optimized the arrangement of stimulus frequencies by making the frequency interval of adjacent stimuli greater than 0.7 Hz and successfully developed a QWERTY-style BCI keyboard with 30 buttons [31]. However, discrepant results have been reported regarding frequency intervals of adjacent stimuli affecting the performance of SSVEP-based BCIs [32]. The number of stimuli was directly proportional to the ITR, and some BCIs with higher ITR have mainly employed an entire keyboard with more stimuli [31]. However, another study described a different outcome: The recognition rate and ITR of SSVEP-based BCIs decreased as the number of stimuli increased [33]. One of the possible reasons for these inconsistent results is the use of different algorithms, which may mask or compensate for the effect of the number of stimuli, resulting in floor effects. Therefore, separate from target recognition algorithms, the effect of the number of stimuli on signal strength and the performance of SSVEP-based BCI are also investigated in this study.

To sum up, it is necessary to examine the independence of SSVEP, in terms of frequency allocation, as it is the assumption behind marking multiple stimuli simultaneously. Furthermore, the independence of SSVEP from number of stimuli can also affect the keyboard design of SSVEP based-BCIs, due to the strength of input signals. Therefore, the purpose of this study was to explore the independence of SSVEP, in terms of frequency allocation and number of stimuli, in order to evaluate the validity of SSVEP as an attention tag and a BCI input signal. It consists of two parts: Experiment 1 aimed to examine the interaction of SSVEP responses under various frequency allocations. Further, Experiment 2 explored whether SSVEP responses were affected by the number of stimuli and compared the performance of SSVEP-based BCIs under different numbers of stimuli.

## 2. Experiment 1: The Independence of SSVEP on Frequency Allocation

### 2.1. Method

#### 2.1.1. Participants

A total of 11 healthy participants (six males, age range = 20–27 years old) without uncorrected visual impairments or any known cognitive deficit participated in the experiment. The experiments were approved by the University of Zhejiang Institutional Review Board. Participants provided their informed consent and were paid for their participation. They were all right-handed and reported normal or corrected-to-normal vision. None reported any history of psychiatric or neurological disorders. 

#### 2.1.2. Stimuli and Procedure

The participants were seated in a comfortable chair 60 cm away from a standard 23.6 inch LCD monitor (SAMSUNG S24E360HL, 60 Hz refresh rate, 1280 × 720 pixels resolution) in a shielded room. Stimuli and tasks were programmed in Python using the PsychoPy libraries [34]. 

The 6° × 6° squares were adopted as stimuli. A letter (A or B) was presented at the center of each square. The font used was Microsoft Yahei Light with font size 20. The letter in the target square was white in the clue phase and turned red in the flashing phase. The square appeared in red in the clue phase and changed to white–black in the flashing phase. The luminance values of the two squares were respectively modulated by sinusoidal coding at a certain frequency, specified as follows:

$$\mathrm{stim}\left(n,f,\varphi \right)=\sin\left(2\pi f\left(\frac{n}{R}\right)+\varphi \right),$$

where n is the serial number of each frame in the stimulus sequence, f is the encoding frequency, *φ* is the encoding phase, and *R* is the refresh rate of the screen. In addition, stim(n, f, *φ*) ranged from −1 to 1, where −1 denotes black and 1 denotes white [35,36].

**Figure 1:**
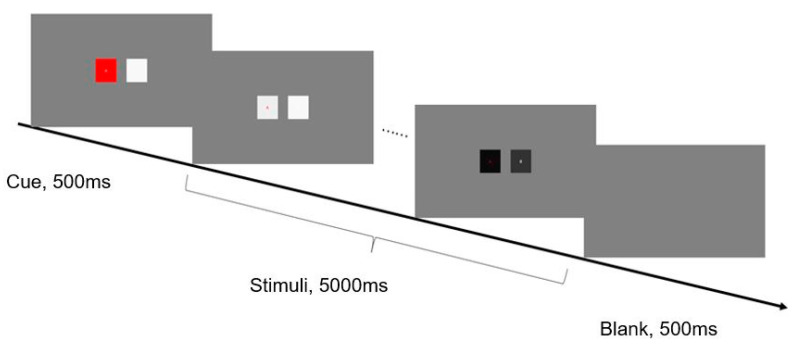
An example sequence of displays in a typical trial of Experiment 1. The experimental stimuli were two squares presented in the center of the screen. Each trial started with a 500 ms cue phase. In this stage, the two squares were at rest, one of which was red (target) and the other was white. Then, the luminance values of two squares were respectively modulated by sinusoidal coding at different frequencies, e.g., alternated from black and white, and they flashed for 5 s. Between trials, an empty screen was presented for 500 ms. Participants were required to pay attention to the red square, where the center letter was the object of fixation.

In Experiment 1, two squares were distributed symmetrically in the center of the screen, 9° apart. The letters A and B were presented, respectively, at the center of each square. A typical selective attention paradigm was adopted. Each trial started with a 500 ms cue phase. In this stage, the two squares were at rest, one of which was red (target) and the other was white. Then, they flashed for 5 s at different frequencies. Between trials, an empty screen was presented for 500 ms. Figure 1 illustrates the detailed timing of one run. During the experiment, there were no explicit behavioral tasks, and participants were instructed to pay attention to the letter in the target square according to the cue (the red square in the cue phase) and avoid unnecessary movement.

It was a complete crossover design, where two flickering frequencies—corresponding to the two stimuli of left and right sides—were selected at 8, 10, 12, 14, 16, 18, 20, 22, and 24 Hz for the two stimuli; that is, 9 frequencies and 81 allocations. There were 81 (frequency allocation) × 3 (repetition) × 2 (pay attention to the left vs. pay attention to the right) = 486 trials. The order of the appearance of each trial was determined randomly. That is, the probability of the target position is 50% on the left or right, that is, the target appeared on the left or right in half of all trials, but the target position was random and fixed in single trial according to the cue (the red square in the cue phase). Additionally, a single stimulus, in which only one square (which flickered at a frequency of 8, 10, 12, 14, 16, 18, 20, 22, and 24 Hz) was presented on the center of the screen, was considered for comparison, with 27 trials in total in an individual test. Ultimately, the duration of Experiment 1 was about 60 min. 

#### 2.1.3. Data Recording and Analysis

EEG was acquired with a 32 Ag/AgCl electrode cap (ESI-32 from Neuroscan), which included standard 10–5 system locations and additional intermediate positions. During EEG data recording, the reference electrode was M1 at the left mastoid. An additional electrode linked to the ground was placed between Fz and FPz, and four other electrodes were used to record the vertical and horizontal eye movements, which were directly connected to the amplifier and placed under the center of each eye and at the outer canthi. The impedance was kept below 5 KΩ for all electrodes. Amplifier bandpass was 0.05–100 Hz and the sampling rate was 1 KHz. Independent component analysis (ICA) assumes that the observed random signal x follows the model x = A×s, where s is unknown source signal whose components are independent of each other, and A is an unknown mixing matrix. The purpose of ICA is to estimate the mixing matrix A and the source signal s by observing x, which is a common method to remove noise from the raw EEG data. Based on independent component analysis (ICA), the components reflecting eye movements and other major artifacts (e.g., the muscle artifact component) were removed for each participant.

EEG data were analyzed with EEGLAB (https://sccn.ucsd.edu/wiki/EEGLAB_References) version 14.1.1 (Swartz Center for Computational Neuroscience, Cambridge, UK) in MATLAB (2017), where the raw data were subjected to 5th-order Butterworth bandpass filtering from 7 to 70 Hz, with the average value of the electrodes on two mastoids serving as a new reference. The data were segmented from 0 to 5000 ms in the flashing phase, and an FFT was performed on the segmented data. As the data length after segmentation was 5000 ms, the spectral resolution after FFT transformation was 0.2 Hz. 

Previous studies have shown that SSVEP has the greatest response at the occipital electrodes [27]. Therefore, the subsequent analysis was based on the average of occipital O1, OZ, and O2 electrodes [31].

In addition to amplitude, another important indicator in spectrum analysis is signal-to-noise ratio (SNR). In the research on SSVEP, the SNR at fn (frequency) is defined as: The ratio of the power of SSVEP at fn to the average power at m surrounding frequencies. However, we mainly analyzed the amplitude of SSVEP in our study. Furthermore, the reason why the SNR results were not reported is that the results of SNR were consistent with the results of amplitude. More important, in the previous studies, researchers always chose either amplitude or SNR to report rather than both.

### 2.2. Results

Statistical analyses were performed by using the SPSS software version 21 (IBM, New York, US). *p*-values <0.05 were considered statistically significant. We first checked whether there were extreme data (excluding data outside of three standard deviations), then examined whether the data were normally distributed and, finally, performed repeated within-subjects analysis of variance (ANOVA).

**Figure 2:**
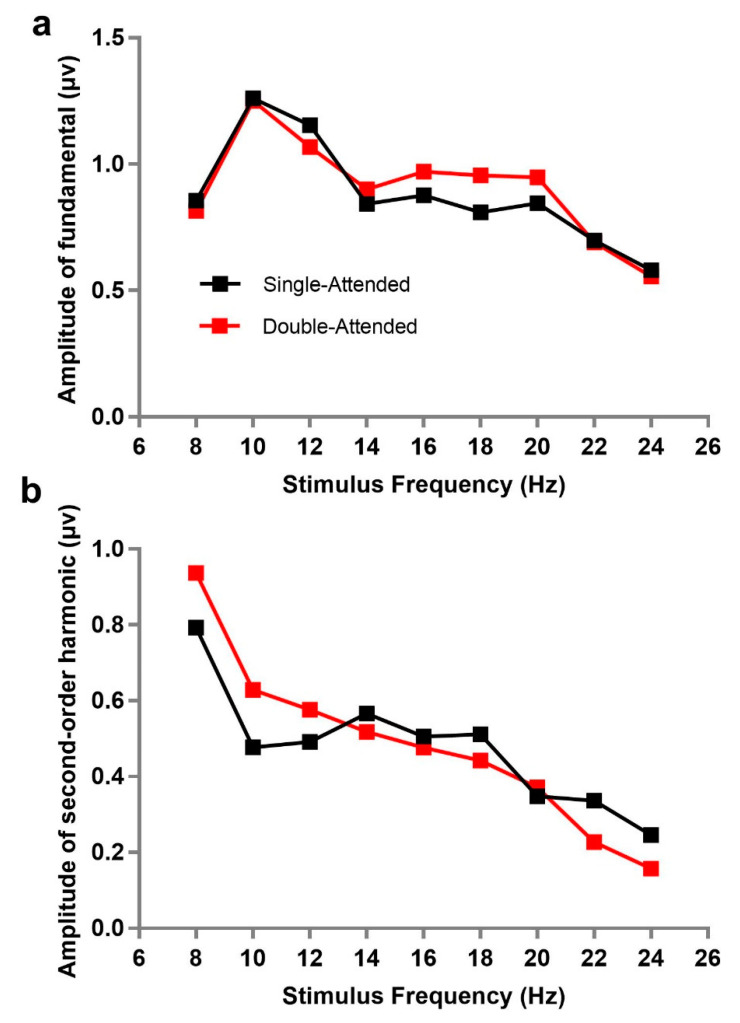
Amplitude change with stimulus frequency under single-attended and double-attended conditions: (**a**) amplitudes of fundamental, and (**b**) amplitudes of second-order harmonic. The horizontal axis shows the flickering frequency of the stimulus and the vertical axis shows the amplitudes corresponding to the stimulus frequency at the fundamental (**a**) and the second-order harmonic (**b**). Single-attended refers to only one stimulus flashing in the central field of vision (as a comparison); double-attended means the target stimulus (attended) was paid attention to while another flashing stimulus was presented simultaneously.

First, we explored the change of SSVEP responses evoked by a stimulus at a certain flickering frequency when another stimulus appeared simultaneously; that is, the amplitudes of SSVEP under single-attended and double-attended conditions were compared at different stimulus frequencies. Single-attended refers to only one stimulus flashing in the central field of vision (as a comparison), while double-attended means a single stimulus (attended) was paid attention to while two stimuli were presented simultaneously at different flickering frequencies. The amplitudes of the fundamental frequencies corresponding to stimuli frequencies were used as the values of SSVEP for data analysis. Subsequently, a 9 × 2 repeated within-subjects analysis of variance (ANOVA) under each stimulus frequency (8, 10, 12, 14, 16, 18, 20, 22, and 24 Hz) and attention condition (single-attended and double-attended) confirmed that the difference of SSVEP amplitudes between single-attended and double-attended (*F*(1, 10) = 0.336, *p* = 0.575, *ƞ_p_*^2^ = 0.033) was absent, as well as the main effects of stimulus frequency (*F*(8, 80) = 1.970, *p* = 0.166, *ƞ_p_*^2^ = 0.165) and their interaction (*F*(8, 80) = 1.269, *p* = 0.295, *ƞ_p_*^2^ = 0.113). Meanwhile, the same analysis of second-order harmonics of SSVEP was performed. It was also found that the second-order harmonic amplitudes under the single-attended condition were not significantly different from those under the double-attended condition (*F*(1, 10) = 0.023, *p* = 0.883, *ƞ_p_*^2^ = 0.002), although the main effect of stimulus frequency (*F*(8, 80) = 16.075, *p* < 0.001, *ƞ_p_*^2^ = 0.616) and their interaction (*F*(8, 80) = 3.542, *p* = 0.030, *ƞ_p_*^2^ = 0.262) were both significant. This implied that there was no gain or loss in the amplitudes of fundamental and second-order harmonics in the attended condition, whether presented separately or with another stimulus. Figure 2 describes the trend of fundamental or second-order harmonic frequency amplitude as a function of stimulus frequency under single-attended and double-attended conditions. We can observe that the second-order harmonic amplitude of SSVEP was larger when the stimulus frequency was lower, suggesting that the magnitude of corresponding frequency may be an important factor for second-order harmonic amplitudes.

To explore the independence of SSVEP on frequency allocation at different frequencies and to analyze directly whether the responses of attended and unattended stimuli were gained or lost under different frequency allocation, a 9 × 9 repeated measures analysis of variance for the fundamental frequency amplitudes with the attended frequency (8, 10, 12, 14, 16, 18, 20, 22, and 24 Hz) and unattended frequency (8, 10, 12, 14, 16, 18, 20, 22, and 24 Hz) was performed. The results demonstrated that their main effects and interactions were all not significant (attended frequency: *F*(8, 80) = 2.057, *p* = 0.160, *ƞ_p_*^2^ = 0.171; unattended frequency: *F*(8, 80) = 0.583, *p* = 0.656, *ƞ_p_*^2^ = 0.055; interaction: *F*(64, 640) = 1.880, *p* = 0.089, *ƞ_p_*^2^ = 0.158), indicating that SSVEP responses at fundamental frequencies under attended and unattended conditions did not significantly change, regardless of the emergence of another stimulus under various frequency allocations. Moreover, a similar analysis was carried out for second-order harmonics, where a significant main effect of attended frequency appeared (*F*(8, 80) = 16.998, *p* = 0.001, *ƞ_p_*^2^ = 0.630), but the main effects of unattended frequency (*F*(8, 80) = 1.016, *p* = 0.408, *ƞ_p_*^2^ = 0.092) and interaction (*F*(64, 640) = 1.390, *p* = 0.234, *ƞ_p_*^2^ = 0.122) were not significant. This indicated that the second-order harmonic amplitudes evoked by attended and unattended stimuli did not interact with each other, but there was a significant difference in the modulation of attention on the second-order harmonics at different stimulus frequencies.

**Figure 3:**
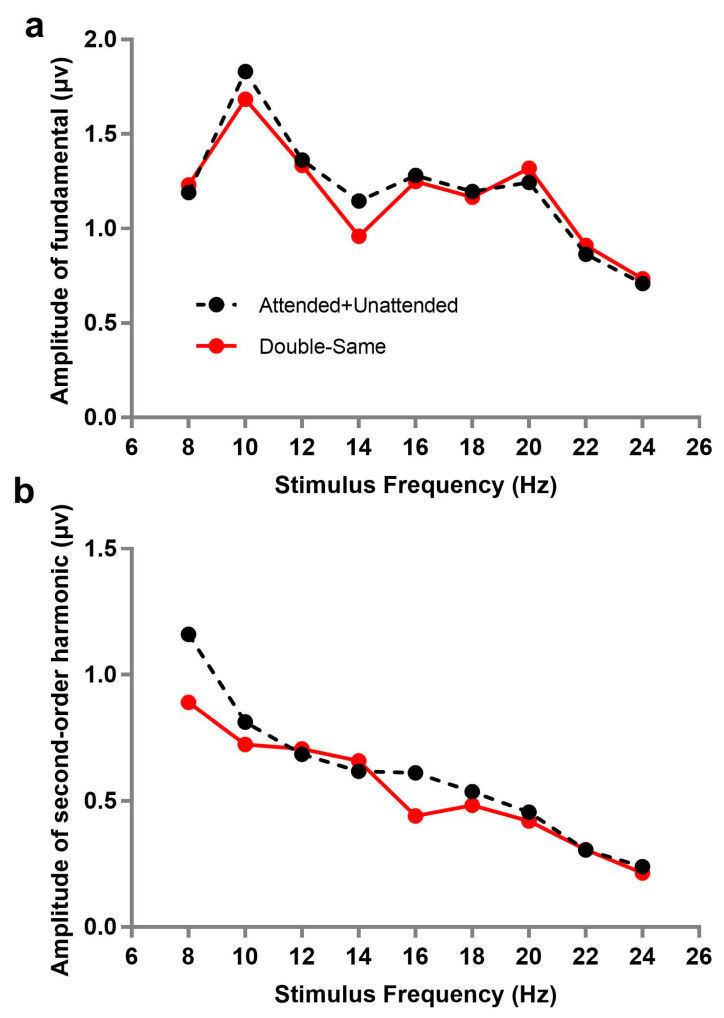
Amplitudes changed with stimulus frequency under different attention conditions: (**a**) amplitudes of fundamentals, (**b**) amplitudes of harmonics. Double-same (co-frequency) refers to two stimuli flashing simultaneously at same frequency; attended + unattended refers to the sum of responses of double-attended and double-unattended (double-attended corresponding to the attended stimulus when two flashing stimuli were presented simultaneously; while double-unattended, to the contrary, corresponds to the unattended stimulus in the same situation).

Additionally, we analyzed the spectrum of SSVEPs under other two attention conditions, double-same and attended + unattended. Double-same (co-frequency) refers to two stimuli flashing simultaneously at same frequency, which cannot be separated from the frequency spectrum; in other words, the amplitude of SSVEP under the double-same condition was the sum of responses evoked by attended and unattended stimuli. Similarly, the amplitude of attended + unattended was the sum of responses of double-attended and double-unattended (double-attended corresponding to the attended stimulus when two flashing stimuli were presented simultaneously; while double-unattended, on the contrary, corresponds to the unattended stimulus in the case). Figure 3 describes the amplitude trend of fundamental or second-order harmonic frequencies as a function of stimulus frequency under the double-same and attended + unattended conditions. A 9 × 2 repeated within-subjects analysis of variance (ANOVA) with each stimulus frequency (8, 10, 12, 14, 16, 18, 20, 22, and 24 Hz) and attention condition (double-same, attended + unattended) on the fundamental and second-order harmonic, respectively, was also performed. The results showed that there was no significant difference of SSVEP responses between double-same and attended + unattended conditions under the fundamental (*F*(1, 10) = 0.347, *p* = 0.569, *ƞ_p_*^2^ = 0.034), but a similar trend failed to appear under second-order harmonic (*F*(1, 10) = 9.637, *p* = 0.011, *ƞ_p_*^2^ = 0.491). This illustrates that the amplitudes of fundamentals in double-same were not significantly different from the sum of double-attended and double-unattended.

## 3. Experiment 2: The Independence of SSVEP on Number of Stimuli

### 3.1. Method

#### 3.1.1. Participants

Participants in Experiment 2 were the same as those in Experiment 1.

#### 3.1.2. Stimuli and Procedure

The experimental material of Experiment 2 was similar to that Experiment 1. 

**Figure 4:**
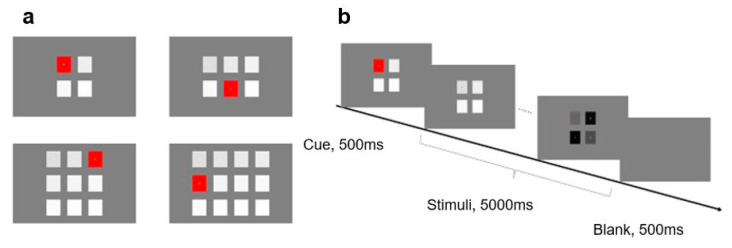
Stimuli and procedure in Experiment 2: (**a**) Four kinds of layout with different numbers of stimuli; and (**b**) an example sequence of displays in a typical trial of Experiment 2.

Differing from Experiment 1, this experiment focused on the effect of the number of stimuli on SSVEP. Accordingly, combined with stimulus frequency, the number of stimuli was manipulated to form various stimulus allocations in a similar frequency range (see Figure 4a). Specifically, when the number of stimuli was 4, the stimulus frequencies were 8.0, 10.0, 12.0, and 14.0 Hz, respectively; when the number of stimuli was 6, the stimulus frequencies were 8.0, 9.4, 10.8, 12.2, 13.6, and 15 Hz, respectively; when the number of stimuli was 9, the stimulus frequencies were 8.0, 8.8, 9.6, 10.4, 11.2, 12.0, 12.8, 13.6, and 14.4 Hz, respectively; when the number of stimuli was 12, the stimulus frequencies were 8.0, 8.6, 9.2, 9.8, 10.4, 11.0, 11.6, 12.2, 12.8, 13.4, 14.0, and 14.6 Hz, respectively. All stimulus frequencies were arranged from left to right and from top to bottom.

The equipment and procedure of Experiment 2 were consistent with that of Experiment 1 (see Figure 4b). According to the position of the target, there were 31 (4 + 6 + 9 + 12) conditions in the whole experiment, where each condition was repeated 6 times for a total of 186 trials. The duration of Experiment 2 was around 20 min.

#### 3.1.3. Data Recording and Analysis

The EEG recording parameters and data analysis method of Experiment 2 were identical to those of Experiment 1. 

In addition, Experiment 2 also included an analysis of the effect of stimulus number on the performances of SSVEP-based BCI. Recognition rate and ITR are two important indicators for assessing BCI performance. Recognition rate was measured with canonical correlation analysis (CCA) [37], which was performed on EEG data recorded by nine electrodes in occipital and temporal regions (P7, P5, P3, POz, P4, P6, O1, Oz, and O2), while ITR (bits per minute) was calculated as follows [23]:

$$ITR=\left(log_{2}M+Plog_{2}P+\left(1-P\right)log_{2}\left(\left(1-P\right)/\left(M-1\right)\right)\right)\times 60/T$$

where *M* is the stimulus number, *P* is the recognition rate, and *T* is the length of time required to focus on the target stimuli.

## 4. Results

**Figure 5:**
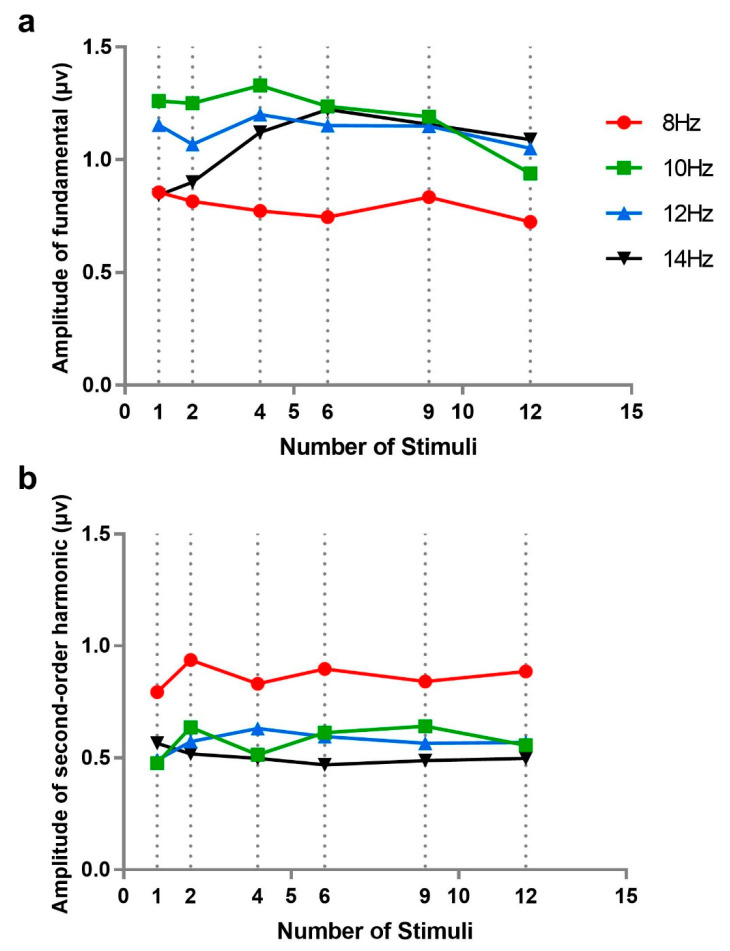
The amplitudes of steady-state visual evoked potentials (SSVEPs) corresponding to four stimulus frequencies (8, 10, 12, and 14 Hz) at six numbers of stimuli (1, 2, 4, 6, 9, and 12): (**a**) the amplitudes of fundamentals, and (**b**) the amplitudes of second harmonics. Due to inconsistent stimulus frequencies across different numbers of stimuli, the amplitudes of nearest frequencies or the averages of two nearest frequencies represented the amplitudes of 10, 12, and 14 Hz.

First, we examined the independence of SSVEP under number of stimuli from the perspective of signal strength by analyzing the amplitudes of fundamentals and second-order harmonics at different numbers of stimuli (see Figure 5). Experiment 2 selected four frequencies of 8, 10, 12, and 14 Hz for comparison. In fact, in order to keep a similar frequency range, stimulus frequencies were not exactly the same across different numbers of stimuli: 10, 12, and 14 Hz were not all used when the number of stimuli is 6, 9, and 12 but were applied with the amplitudes of nearest frequencies or the averages of two nearest frequencies. 

A 4 × 6 repeated measures analysis of variance was performed on fundamental frequency amplitudes with stimulus frequency (8, 10, 12, and 14 Hz) and number of stimuli (1, 2, 4, 6, 9, and 12; the data for 1 and 2 stimuli were taken from Experiment 1). The results showed that main effects of stimulus number (*F*(5, 50) = 0.449, *p* = 0.641, *ƞ_p_*^2^ = 0.043) and stimulus frequency (*F*(3, 30) = 2.372, *p* = 0.123, *ƞ_p_*^2^ = 0.192) and their interaction (*F*(15, 150) = 1.449, *p* = 0.251, *ƞ_p_*^2^ = 0.127) were not significant. Furthermore, we analyzed the amplitude of fundamentals separately in the case of 8 Hz, in order to eliminate the influence of frequency interpolation; however, the effect of stimulus number was still not significant (*F*(5, 50) = 0.252, *p* = 0.819, *ƞ_p_*^2^ = 0.025). This indicated that there was no difference in the amplitude of fundamentals among different numbers of stimuli.

The same analysis was performed for the second-order harmonic amplitudes of SSVEP. A similar result was found: the main effects of number of stimuli (*F*(5, 50) = 0.227, *p* = 0.795 *ƞ_p_*^2^ = 0.027) and interaction (*F*(15, 150) = 0.877, *p* = 0.485, *ƞ_p_*^2^ = 0.081) were not significant. This indicated that there was no notable difference in the amplitude of harmonics among different numbers of stimuli. However, the main effect of stimuli frequency (*F*(3, 30) = 13.537, *p* = 0.001, *ƞ_p_*^2^ = 0.575) was significant, which showed that there were obvious differences among the second-order harmonics of different stimulus frequencies, consistent with the results of Experiment 1. Additionally, the effect of number of stimuli in the case of 8 Hz was tested separately, which was still not significant (*F*(5, 30) = 0.356, *p* = 0.749, *ƞ_p_*^2^ = 0.034). 

**Figure 6:**
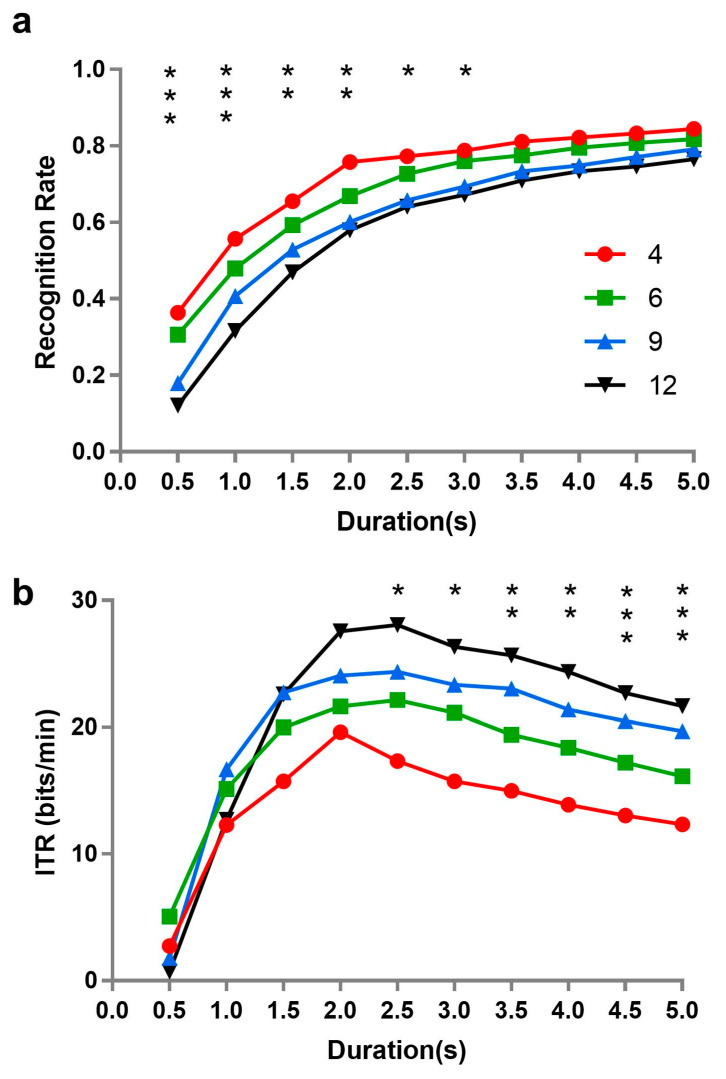
The performance of SSVEP-based brain–computer interface (BCI) for different numbers of stimuli and time lengths: (**a**) recognition rate, and (**b**) information transfer rate (ITR). The asterisk “*” indicates a significant difference among different stimulus numbers (i.e., * *p* < 0.05, ** *p* < 0.01, *** *p* < 0.001).

Meanwhile, we compared the performance of a SSVEP-based BCI under the same algorithm and different numbers of stimuli. To our knowledge, the duration of inputs is an important factor for the performance of BCI. The recognition rate of the SSVEP-based BCI was, therefore, separately identified by the algorithm of CCA under different durations when the number of stimuli was 4, 6, 9, and 12, with the ITR respectively computed, as well. The independence of SSVEP from the number of stimuli was further tested under different durations, from the perspective of the performance of the BCI. Figure 6 describes the recognition rate and ITR corresponding to 4, 6, 9, and 12 stimuli at different temporal lengths.

Repeated measures analysis of variance was performed on recognition rate with time length (0.5, 1, 1.5, 2, 2.5, 3, 3.5, 4, 4.5, and 5 s) and number of stimuli (4, 6, 9, and 12). All effects were found to be significant (time length: *F*(9, 90) = 71.188, *p* = 0.001, *ƞ_p_*^2^ = 0.877; number of stimuli: *F*(3, 30) = 9.388, *p* = 0.0001, *ƞ_p_*^2^ = 0.484; interaction: *F*(27, 270) = 2.705, *p* = 0.031, *ƞ_p_*^2^ = 0.213). Simple effects were further analyzed, revealing that there were significant differences of recognition rate among different numbers of stimuli when the length of time was short; specifically, the fewer number of stimuli presented at the same time, the higher recognition rate of BCI. However, the number of stimuli did not affect the recognition rate when the time length of stimulus presentation was longer than 3 s.

A similar analysis of ITR revealed that the main effects of time length (*F*(9, 90) = 8.616, *p* = 0.004, *ƞ_p_*^2^ = 0.463) and stimulus number (*F*(3, 30) = 4.140, *p* = 0.029, *ƞ_p_*^2^ = 0.293), as well as their interaction (*F*(27, 270) = 2.491, *p* = 0.055, *ƞ_p_*^2^ = 0.199) were all significant. Simple effect analysis of ITR was also performed. Coincidentally, the results were almost the reverse of those of recognition rate. No significant differences of ITR appeared among different stimulus numbers when length of time was shorter than 2.5 s.

Additionally, the ITR is inversely proportional to recognition rate, but directly proportional to the length of time (duration). We could observe that the recognition rate tended to be stable when the time length of stimulus presentation was longer than 3 s in our results. As the duration of stimulus presentation increases, absolutely, the ITR showed that a trend of decline emerged after 3 s.

## 5. Discussion 

### 5.1. The Mechanism of SSVEP

To our knowledge, SSVEP has been considered to reflect the non-linear characteristics of brains for a long time [29,38]. The non-linearity of SSVEP is mainly reflected in two aspects: first, with the change of stimulus frequency, obvious resonance phenomenon can turn up near 10, 20, and 40 Hz; second, SSVEP has harmonic or inter-modulation frequency components [2]. Recently, it has been found that SSVEP may have both linear and non-linear characteristics, as described in some studies [39,40]. Our study also verified such hybrid characteristics. On one hand, it was observed that obvious resonance phenomena appeared at 10 and 20 Hz of the fundamental frequency (see Figure 2). On the other hand, a comparative analysis of SSVEP responses showed that the amplitudes under the condition of double-same were equal to the linear sum of those under the double-attended and double-unattended, which indicated that the superposition principle could be applied in the amplitudes of fundamentals.

Meanwhile, there was a distinctly different finding between fundamentals and second-order harmonics—that is, as the stimulus frequency increased, the corresponding harmonic responses decreased—while the fundamental responses conformed to the source distribution of the three SSVEP sub-systems (peaks near 10, 20, and 40 Hz) [27]. Compared with the corresponding fundamental frequency band (8–24 Hz), the corresponding second-order harmonic frequency band (16–48 Hz) was higher and wider. As the sensitivity of SSVEP depends on the corresponding response frequency, SSVEP amplitudes at a higher response frequency are always smaller and less stable [41]. This indicates that the sub-system of SSVEP should mainly focus on the response frequency, not the stimulus frequency.

### 5.2. The Validity of SSVEPs as Attention Tags

Numerous studies have explored the generation mechanisms and source locations of SSVEP, but have not reached a consensus [27]. It is widely believed that SSVEPs at different frequency bands originate from different brain regions and involve different visual pathways [42]. At present, it is popular to use SSVEPs as attention tags, in order to explore the effectively dynamic mechanisms of mental processes in cognitive studies [43,44,45]. Hence, a question arises: Are there interactions between two stimuli when they are presented simultaneously at different frequency bands (or the same bands)? In fact, almost all SSVEP-related cognitive studies have been based on some hypotheses. Our study tested one of these hypotheses: the independence of SSVEPs. Specifically, we compared the responses of SSVEP under different attention conditions and in different frequency allocations. 

Regarding the fundamental, first, it was found that the SSVEPs induced by the same frequency stimuli had no difference between the condition of single-attended and double-attended. Second, SSVEPs evoked by the attended stimulus and the unattended stimulus did not have mutual interference when the two stimuli were flashing at the same time at different frequencies. Third, the responses of SSVEP in the double-same condition were almost equivalent to the sum of those in the double-attended and double-unattended conditions. In general, it can be speculated that the fundamental amplitudes of SSVEP induced by different stimulus frequencies are independent; that is, the fundamental amplitude of SSVEP induced by a stimulus flickering at a certain frequency is not disturbed by the presence or absence of other stimuli with different frequencies. It had no effect under frequency allocations. Meanwhile, the independence of SSVEP was also partially reflected in the second-order harmonics. Harmonic responses of SSVEPs evoked by the same stimulus frequency showed no difference between the condition of single-attended and double-attended; SSVEPs at harmonic frequency evoked by the attended or unattended stimulus did not have a mutual interference, either.

In short, the independence of SSVEPs under frequency allocation reflected in fundamentals and second-order harmonics was shown, and no significant gain or loss in SSVEP amplitude appeared among any frequency allocations. Thus, we believe that SSVEP can serve as a tool to mark and monitor multiple stimuli simultaneously, breaking through the bottleneck of cognitive research and making up for the shortcomings of usual ERP. 

### 5.3. The Signal Strength and Performance of SSVEP-Based BCIs

Due to the limitation of available frequency bands and the demand for a large number of inputs, the independence of SSVEP in terms of the number of stimuli has become an indispensable factor for SSVEP-based BCIs [27,31]. We explored the interaction of SSVEP under six numbers of stimuli (1, 2, 4, 6, 9, and 12). There was no difference in SSVEP fundamental and harmonic amplitude responses, no matter how many stimuli were presented at the same time. In other words, in the aspect of signal strength, the independence of SSVEP was embodied in all numbers of stimuli, and SSVEP was shown to be trustworthy as a signal source for BCI.

Recognition rate refers to the probability that a target is identified from many interferences by users, while ITR denotes the amount of information transferred by the system in a unit of time. They are both important indicators for assessing BCI performance [46,47]. In fact, the number of stimuli had no effect on the signal strength but did have an effect on the BCI performance, to a certain degree. The effect of the number of stimuli on recognition rate was relative to the time length of stimulus presentation. Our evidence showed that the recognition rate of BCIs was not affected by the number of stimuli in the case of long duration (longer than 3 s). Nevertheless, in previous studies, most BCIs with high performance had an adequate duration for signal input [30,48]. Moreover, according to the calculation formula of ITR [23], the number of stimuli and the recognition rate could predict ITR in the opposite direction. A similar analysis of recognition rate was performed and, coincidentally, the results were almost the reverse of those for recognition rate. No significant differences of ITR appeared among different numbers of stimuli when the length of time was shorter than 2.5 s.

In short, the independence of recognition rate and ITR of SSVEP, in terms of the number of stimuli, changed among different stimuli durations. Specifically, the recognition rate was not affected by the number of stimuli when the length of time was long enough, and no significant differences appeared in the ITR among different numbers of stimuli when the length of time was short enough. To our knowledge, the number of stimuli was in positive correlation with ITR. Therefore, it is still important to think seriously about the selection of the number of stimuli and duration to achieve the best performance of SSVEP-based BCIs in the future.

### 5.4. Prospects for the Future Studies

Indeed, there are some problems in this study, which need to be solved in the following studies. First, only 11 volunteers participated in the experiments. Although we have paid more attention on the individual data of each subject, and found a highly consistent trend in results, more participants are needed in more experiments to verify our results repeatedly in the future. Second, our study mainly focused on the amplitude of SSVEP, but when it comes to the analysis of wave phenomena, phase difference would be another important variable that needs more concern. The future studies should consider the phase into the validity of SSVEPs as attention tags and input signals as well. Finally, although SSVEPs were adopted in numerous studies, the mechanism of SSVEPs is still unclear. It is worth exploring whether the SSVEPs affect the cognitive process, and more experiments are needed to explore its essence in the future.

## 6. Conclusions

The present study explored the independence of SSVEP, in terms of the frequency allocation and the number of stimuli. Our results revealed that there was no significant gain or loss of SSVEP amplitude under different frequency allocations. Additionally, the signal strength of SSVEP was not affected by the number of stimuli. However, it was shown that the performance of SSVEP-based BCIs varies with the duration of stimuli; that is, the recognition rate was not affected by the number of stimuli when the duration of stimuli was long enough, while information transfer rate (ITR) had an opposite trend.

It was concluded that the independence of SSVEP from frequency allocation can be assumed, and that SSVEP is reliable in marking multiple stimuli with different frequencies simultaneously. The independence of signal strength of SSVEP was embodied in all numbers of stimuli considered, and SSVEP was shown to be trustworthy as an effective signal source for BCIs. However, much care should be taken when choosing a suitable duration and number of stimuli, in order to achieve the optimal utility of SSVEP-based BCIs. 

## Open Practices Statement

The data, materials, and program codes for all experiments are available at http://dx.doi.org/10.17632/yn2nv7b7n2.1.
